# Iodine-Biofortified Microgreens as High Nutraceutical Value Component of Space Mission Crew Diets and Candidate for Extraterrestrial Cultivation

**DOI:** 10.3390/plants12142628

**Published:** 2023-07-12

**Authors:** Maria Giordano, Michele Ciriello, Luigi Formisano, Christophe El-Nakhel, Antonio Pannico, Giulia Graziani, Alberto Ritieni, Marios C. Kyriacou, Youssef Rouphael, Stefania De Pascale

**Affiliations:** 1 Dipartimento di Agricoltura, Alimentazione e Ambiente (Di3A), University of Catania, 95123 Catania, Italy; 2 Department of Agricultural Sciences, University of Naples Federico II, 80055 Portici, Italy; michele.ciriello@unina.it (M.C.); luigi.formisano3@unina.it (L.F.); christophe.elnakhel@unina.it (C.E.-N.); antonio.pannico@unina.it (A.P.); depascal@unina.it (S.D.P.); 3 Department of Pharmacy, University of Naples Federico II, 80131 Naples, Italy; giulia.graziani@unina.it (G.G.); alberto.ritieni@unina.it (A.R.); 4 Department of Vegetable Crops, Agricultural Research Institute, 1516 Nicosia, Cyprus; mkyriacou@ari.moa.gov.cy

**Keywords:** carotenoids, controlled environmental agriculture, iodine deficiencies, polyphenols, secondary metabolites, soilless cultivation, space agriculture

## Abstract

The success of Space missions and the efficacy of colonizing extraterrestrial environments depends on ensuring adequate nutrition for astronauts and autonomy from terrestrial resources. A balanced diet incorporating premium quality fresh foods, such as microgreens, is essential to the mental and physical well-being of mission crews. To improve the nutritional intake of astronaut meals, two levels of potassium iodide (KI; 4 µM and 8 µM) and an untreated control were assessed for iodine (I) biofortification, and overall nutraceutical profile of four microgreens: tatsoi (*Brassica rapa* L. subsp. narinosa), coriander (*Coriandrum sativum* L.), green basil, and purple basil (*Ocimum basilicum* L.). A dose-dependent increase in I was observed at 8 µM for all species, reaching concentrations of 200.73, 118.17, 93.97, and 82.70 mg kg^−1^ of dry weight, in tatsoi, coriander, purple basil, and green basil, respectively. Across species, I biofortification slightly reduced fresh yield (–7.98%) while increasing the antioxidant activity (ABTS, FRAP, and DPPH). LC–MS/MS Q extractive orbitrap analysis detected 10 phenolic acids and 23 flavonoids among microgreen species. The total concentration of phenolic acids increased (+28.5%) in purple basil at 8 µM KI, while total flavonoids in coriander increased by 23.22% and 34.46% in response to 4 and 8 µM KI, respectively. Both doses of KI increased the concentration of total polyphenols in all species by an average of 17.45%, compared to the control.

## 1. Introduction

The Space missions planned in the coming years will allow humans to surpass Earth’s orbit by landing on different planets (e.g., Mars) and returning to the Moon [1]. To ensure the success of these missions, it will be pivotal to provide the crew with supplies of fresh food with high nutritional and nutraceutical value, which are crucial for human adaptation to the adverse conditions of Space environments [2,3]. However, a long-term diet (months or years) based solely on processed foods from Earth can lead to detrimental nutrient deficiencies [4]. Therefore, it is necessary to design an efficient system for on-board production of fresh plant-based food using the latest advanced agricultural technologies [5]. Space agriculture requires food production in cultivation systems with limited use of resources in terms of energy, volume, and mass, maximizing those available on-site such as light, water, and fertilizers, while addressing new environmental challenges such as altered gravity, ionizing radiation, reduced atmospheric pressure, and high CO_2_ levels [1]. Crops for Space cultivation must possess specific characteristics such as a short growth cycle, small size, high yield, and, above all, premium nutritional properties [6]. Several authors have proposed microgreens as potential candidates for Space agriculture because they are easy to manage, allow optimal use of space, are rich in useful phytochemicals, and require limited investment in time and resources for the crew [7,8,9]. Due to their interesting nutritional properties, microgreens are gaining increasing interest and popularity among modern consumers, who are becoming more aware of their dietary choices [10]. Due to their rapid growth and positive impact on human health (high bioavailability of polyphenols, glucosinolates, vitamins, and minerals), microgreens are considered useful ‘superfoods’ to enrich and diversify the diet. They could also represent a healthy garnish for future astronauts, as they have the potential to counteract the underlying nutritional deficiencies that often affect humans both on land and in Space, leading to chronic diseases [11,12]. For example, secondary metabolites such as ascorbic acid are potent radiomitigators capable of mitigating the potential damage from ionizing radiation [13]. A wide variety of species belonging to different botanical families can be grown as microgreens, and, consequently, more and more scientific studies are investigating in detail the effect of genotype on yield and quality [14,15,16]. In addition to genetic material, pre-harvest factors such as light, temperature, nutritional management, plant phenological stage at the time of harvest, cultivation density, and substrate used strongly influence the growth parameters and secondary metabolism of microgreens [17,18,19]. 

A promising agronomic strategy to improve the nutraceutical profile of vegetables, including microgreens, is biofortification, which contributes to the achievement of the recommended daily levels of essential micronutrients. Among micronutrients, iodine is particularly important for humans, as it is a component of the hormone triiodothyronine and thyroxine, and is involved in the regulation of numerous cellular processes, including protein synthesis and nucleic acid metabolism, fats, and sugars [20,21]. The daily requirement of iodine varies according to age, ranging from 90 μg day^−1^ for children (up to 8 years old) to 270 μg day^−1^ for breastfeeding women [22]. For example, iodine deficiency during pregnancy can affect fetal development or cause physical and mental damage to the unborn child [23]. The main dietary sources of iodine are fish, seaweed, milk, and dairy products [24,25], while fruits and vegetables contain relatively low amounts [24,25]. Based on this, the cultivation of iodine-biofortified microgreens could be the key to integrating the astronauts’ diet. 

To date, there are few studies that have thoroughly investigated the effects of iodine biofortification on the physiological traits and phytochemical properties of microgreens, since more attention has been paid to selenium biofortification [26]. The present research paper constitutes a continuation of our previously published work on agronomic biofortification with iodine of four different species of microgreens and its implications for mineral profile and contribution to the RDI of this essential micronutrient [27]. Based on these considerations, the present study investigated the effects of biofortification with different levels of iodine (4 µM and 8 µM) on the nutraceutical and biochemical characteristics of three families of microgreens [Brassicaceae (tatsoi, *Brassica rapa* L. subsp. narinosa), Apiaceae (coriander, *Coriandrum sativum* L.), and Lamiaceae (green and purple basil, *Ocimum basilicum* L.)].

## 2. Results

### 2.1. Microgreens Yield

Fresh biomass of the four microgreen genotypes examined is statistically different (Table 1), with the highest being attributed for coriander (1.78 kg m^−2^), followed by tatsoi (1.52 kg m^−2^), green basil (1.10 kg m^−2^), and purple basil, which had the lowest values (1.04 kg m^−2^). Similarly, coriander had the highest dry biomass values (0.20 kg m^−2^) and purple basil the lowest (0.09 kg m^−2^). Tatsoi also had the shorter hypocotyl (3.17 cm), compared to coriander, purple basil (4.20 and 4.52, respectively), and green basil (3.76 cm). The iodine treatment had different effects on the fresh biomass of the four microgreen species (S × B). The fresh biomass of tatsoi was reduced with the 4 µM iodine dose (−13.12%), while coriander biomass is reduced (−8.65%) with the 8 µM dose, compared to the control. The fresh biomass of green basil was reduced (−11.66% on average) with both levels of iodine, compared to the control. The fresh biomass of purple basil does not change with iodine treatments, compared to the control. Regardless of the microgreen species, iodine treatment reduced dry biomass (−7.7%), compared to the control. The hypocotyl length of purple basil was reduced by an average of 9.8%, compared to the control, with both doses of iodine.

### 2.2. Iodine Biofortification

**Figure 1:**
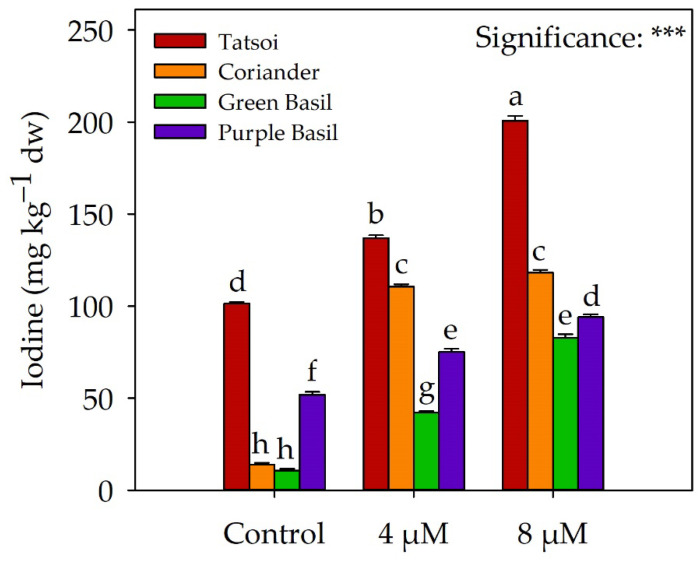
Effect of species × iodine biofortification interaction on the iodine concentration of four genotypes of microgreens. Different letters above the bars indicate significant differences among the genotypes according to Tukey’s HSD test (*p* = 0.05). *** significant at *p* ≤ 0.001. All data are expressed as mean ± SE, *n* = 3. dw: dry weight.

As shown in Figure 1, tatsoi was the species with the highest iodine concentration, with a value of 101.30 mg kg^−1^ dw. Purple basil had approximately half the iodine of tatsoi, with a value of 51.83 mg kg^−1^ dw. On the other hand, coriander and green basil had a 10-fold lower iodine concentration than tatsoi, with values of 13.90 mg kg^−1^ dw and 10.80 mg kg^−1^ dw, respectively. Iodine accumulated significantly in microgreens as the applied dose increased. In tatsoi, the iodine concentration reached values of 137 and 200.73 mg kg^−1^ dw with doses of 4 and 8 µM, respectively; in green basil, 42.17 and 82.70 mg kg^−1^ dw, respectively, and purple basil, 75.03 and 93.97 mg kg^−1^ dw, respectively. In coriander, the iodine level reached values of 110.57 and 118.17 mg kg^−1^ dw with 4 and 8 µM of iodine in the nutrient solution, respectively, without significant differences between the two doses. 

### 2.3. Pigments Concentration and Antioxidant Activity

**Figure 2:**
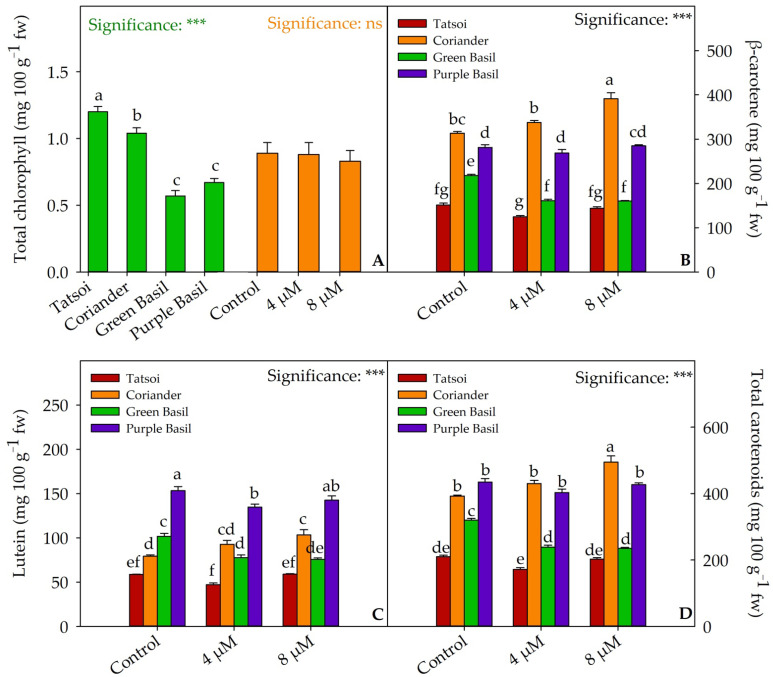
Mean effects of species and iodine biofortification ((**A**), green and orange bars, respectively) on chlorophyll concentration and effect of species × iodine biofortification interaction on β-carotene (**B**), lutein (**C**), and total carotenoids (**D**) concentration of four microgreens species. Different letters above the bars indicate significant mean differences among the genotypes according to Tukey’s HSD test (*p* = 0.05). *** significant at *p* ≤ 0.001. All data are expressed as mean ± SE, *n* = 3. fw: fresh weight.

**Figure 3:**
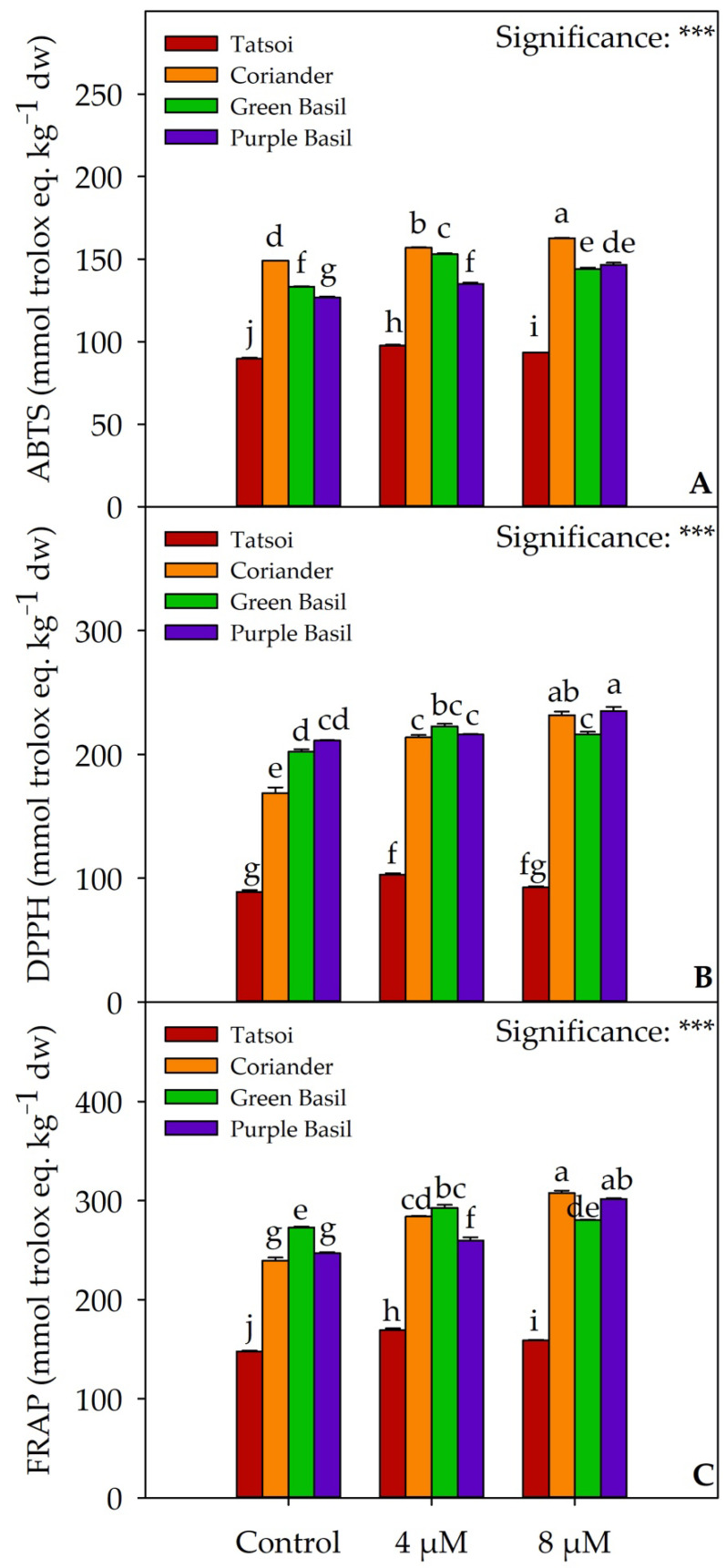
Effects of species × biofortification interaction on ABTS (**A**), DPPH (**B**), and FRAP (**C**) antioxidant activities of four microgreens species. Different letters above the bars indicate significant mean differences within each genotype according to Tukey’s HSD test (*p* ≤ 0.05). *** significant at *p* ≤ 0.001. All data are expressed as mean ± SE, *n* = 3. dw: dry weight.

Tatsoi microgreens had the highest total chlorophyll concentration (1.20 mg 100 g^−1^ of fresh weight (fw)), followed by coriander (1.04 mg 100 g^−1^ fw), while green basil and purple basil had the lowest concentrations (0.57 and 0.67 mg 100 g^−1^ fw, respectively; Figure 2A and Supplementary Table S1). Statistical differences were also found for the carotenoids concentrations (Figure 2B–D and Supplementary Table S1). β-carotene had the highest concentration in coriander (313.38 mg 100 g^−1^ fw), followed by purple basil (281.17 mg 100 g^−1^ fw), and green basil (218.12 mg 100 g^−1^ fw; Figure 2B and Supplementary Table S1). Purple basil had the highest values of lutein (153.18 mg 100 g^−1^ fw), followed by green basil (101.7 mg 100 g^−1^ fw) and coriander (79.23 mg 100 g^−1^ fw; Figure 2C and Supplementary Table S1). The total carotenoid values were higher in purple basil and coriander (434.34 and 392.61 mg 100 g^−1^ fw, respectively), followed by green basil (319.82 mg 100 g^−1^ fw; Figure 2D and Supplementary Table S1). Tatsoi had the lowest values of β-carotene, lutein, and total carotenoids (151.11, 58.73, and 209.84 mg 100 g^−1^ fw, respectively). Iodine treatment did not change the carotenoids concentration in tatsoi microgreens, compared to the control. In coriander, the 8 µM dose increased the concentration of β-carotene, lutein, and total carotenoids by 24.9%, 30.27%, and 26%, respectively, compared to the control. An opposite effect was obtained in green basil, in which both doses of iodine reduced carotenoids, compared to the control. In purple basil, the 4 µM dose reduced the lutein concentration (−12%), compared to the control. Coriander was the species with the highest antioxidant activity measured by the ABTS method (149.02 mmol Trolox equivalents kg^−1^ dw; Figure 3A and Supplementary Table S1). Purple basil (211.33 mmol Trolox equivalents kg^−1^ dw) and green basil (272.72 mmol Trolox equivalents kg^−1^ dw) had higher antioxidant activity measured by the DPPH and FRAP methods, respectively (Figure 3B,C and Supplementary Table S1). Tatsoi, among the four species tested, had the lowest antioxidant profile measured by the three methods. Both iodine treatments had an increased antioxidant activity measured with the three methods (Supplementary Table S1).

### 2.4. Phenolic Acids

The Q Exactive Orbitrap LC–MS/MS analysis revealed the presence of 10 phenolic acids among the four species of microgreens examined. Green basil and purple basil had the highest total phenol acid values (14,744.46 µg 100 g^−1^ fw and 14,023.23 µg 100 g^−1^ fw, respectively), followed by coriander (4278.96 µg 100 g^−1^ fw), while tatsoi had the lowest values (269.33 µg 100 g^−1^ fw). Regardless of iodine treatment, tatsoi was the microgreens species with the highest values of caffeic acid hexoside (69.25 µg 100 g^−1^ fw), coumaroyl quinic acid (16.92 µg 100 g^−1^ fw), and sinapinic acid hexose (161.79 µg 100 g^−1^ fw). Coriander had the highest values of feruloyl quinic acid (607.27 µg 100 g^−1^ fw), caffeoyl quinic acid (3574.32 µg 100 g^−1^ fw), and ferulic acid (65.76 µg 100 g^−1^ fw). In these two species of microgreens, three phenolic acids (rosmarinic acid, caffeoyl–feruloyl–tartaric acid, and cichoric acid) were not detected, but were instead detected in green basil (12,780.35 µg 100 g^−1^ fw, 355.25 µg 100 g^−1^ fw, 1431.68 µg 100 g^−1^ fw, respectively) and purple basil (12,446.82 µg 100 g^−1^ fw, 208.06 µg 100 g^−1^ fw, and 1239.51 µg 100 g^−1^ fw, respectively). The two iodine treatments did not produce significant effects on the total phenolic acid concentration in tatsoi, coriander, and green basil, compared to the untreated control. On the other hand, in purple basil, the 8 µM dose increased total phenolic acids by 28.5%, compared to the control. The two doses of iodine increased the concentration of caffeic acid hexoside (+34.65% on average), coumaroyl quinic acid (+43.61% on average), and sinapinic acid hexose (+15.07% at a dose of 4 µM) in tatsoi; feruloyl quinic acid (+13.64% on average) in coriander; and the concentration of caffeic acid in green basil (+46.75% on average), compared to the control (S × B interaction, Table 2). In both basil species, the two iodine treatments increased the rosmarinic acid concentration by an average of 22.24%, compared to the control. 

### 2.5. Total Polyphenols and Flavonoids

Regardless of treatment, green basil and purple basil had the highest polyphenolic concentrations (16,473.54 µg 100 g^−1^ fw and 15,849.61 µg 100 g^−1^ fw, respectively), followed by coriander (10,970.03 µg 100 g^−1^ fw) and tatsoi (480.44 µg 100 g^−1^ fw). Regardless of the microgreen species, iodine treatment increased the total polyphenol concentration by 17.45% on average, compared to the control (Table 3). Coriander was found to be particularly rich in total flavonoids with a value of 5542.3 µg 100 g^−1^ fw, while in tatsoi, purple basil, and green basil had much lower values (175.8, 117.85, and 57.44 µg 100 g^−1^ fw, respectively). The two iodine treatments did not show a significant effect on total flavonoids in tatsoi, green, and purple basil, while in coriander this value increased by 23.22% with the 4 µM dose and by 34.46% with the 8 µM dose. Of the 23 flavonoids, not all were detected in the four species of microgreens. Regardless of the iodine treatment, the most abundant flavonoid in tatsoi is km 3-synapoyl-sophoroside-7-glucoside with the value of 95.04 µg 100 g^−1^ fw. Coriander distinguished itself from other species for its high rutin concentration (5373.4 µg 100 g^−1^ fw). In green basil, the most abundant flavonoid was apigenin-7-rutinoside (15.55 µg 100 g^−1^ fw), and in purple basil it was km 3-p-coumaroylsophoroside-7-glucoside (34.67 µg 100 g^−1^ fw). 

Flavonoid concentration was significantly influenced by dose and genotype (S × B). In tatsoi, the 8 µM dose increased Km 3-O-caffeoyl-spophoroside-7-glucoside (+34.6%), compared to the control, while the same dose reduced luteolin-malonyl-hexose (−53%). Furthermore, in tatsoi both doses reduced km 3-synapoyl-sophoroside-7-glucoside, compared to control. In coriander, 8 µM increased the concentration of km 3-sophoroside-7-glucoside (+192.72%), and luteolin-7-rutinoside (+27.86%), compared to the control, and both doses increased rutin (+23.88% with 4 µM, and +35.12% with 8 µM). Both doses of iodine increased apigenin-7-rutinoside (+29.1% on average) in green basil and reduced apigenin-malonyl-glucoside, in green and purple basil, compared to control treatment (Table 3).

## 3. Discussion

### 3.1. Effect of Iodine Biofortification on Yield and Growth Parameters

In our study, we observed that tatsoi (*Brassica rapa* L. subsp. Narinosa) microgreens had a faster growth cycle, reaching maturity in 14 days after sowing (DAS), compared to coriander (*Coriandrum sativum* L.), green basil, and purple basil (*Ocimum basilicum* L.) microgreens, which take 21 DAS to reach maturity. This finding aligns with a similar study conducted by Kyriacou et al. [15], where tatsoi also exhibited a shorter growth cycle of 16 DAS, while coriander and basil microgreens took 19–20 days to reach maturity. However, the fresh biomass values reported by Kyriacou et al. [15] were higher than those found in our study, with green basil, purple basil, tatsoi, and coriander microgreens achieving fresh biomass of 1.62, 3.09, 3.16, and 3.30 kg m^−2^ fw, respectively. The disparity in fresh biomass between the two studies could be attributed to the different growing mediums used. In our study, we utilized a chemically inert material, while Kyriacou et al. [15] grew their microgreens on peat, which provides optimal ventilation, moisture for root growth, and a supply of nutrients. 

Furthermore, in our study, the response of microgreens to iodine treatment in terms of fresh biomass was dependent on the genotype and dosage of iodine (Table 1). Similar observations were reported in biofortification studies with iodine in mature species, where the response to iodine supplementation varied depending on the species, iodine dosage, and experimental conditions. For instance, Golob et al. [28] found that an 8 mM I foliar treatment on kohlrabi (*Brassica oleracea* var. Gongylodes) had no toxic effects on photosynthetic efficiency, morphological properties, or plant yield. Likewise, studies on spinach [29], four Brassica species [30], and lettuce (*Lactuca sativa* L.) [31] reported no toxic effects on yield reduction, necrosis, or chlorosis when different levels or forms of iodine were applied. However, Blasco et al. [32] observed toxicity in lettuce when irrigated with potassium iodide (KI) concentrations ranging from 10 to 240 µmol L^−1^. Biomass reduction was observed starting from a concentration of 40 µM I^−^ in the nutrient solution, and this phytotoxic effect was attributed to the accumulation of iodine in plant tissues or its oxidation to I_2_, which interfered with photosynthesis. 

In the study by Blasco et al. [33], lettuce (var. longifolia) plants were grown in a growth chamber and irrigated with a nutrient solution containing different doses of potassium iodide (KI) (20, 40, 80 µM), compared to a control group that received an iodine supplement-free solution. The researchers found that at doses of 20 and 40 µM, there was an increase in stomatal conductance and a slight increase in photosynthetic activity. However, these changes did not result in a significant increase in biomass compared to the control group. It is possible that the photosynthetic products, such as glucose and fructose, were utilized by the plant for various physiological functions or accumulated as starch in the chloroplasts. The authors noted that the dose of 80 µM reduced stomatal conductance and transpiration, leading to a reduction in photosynthetic activity and biomass. This higher dose of iodine negatively affected the plants’ physiological processes and resulted in decreased growth. In another study by Blasco et al. [34], it was demonstrated that iodine (I^−^) treatment in lettuce leaves reduced the absorption of nitrate, which could be attributed to alterations in the transporters or an antagonistic effect between anions. This reduction in nitrate absorption resulted in decreased total nitrogen accumulation (TNA) and nitrogen uptake efficiency (NupE). However, the nitrogen utilization efficiency (NutE), which represents the efficiency of translocating nitrogen to the shoots, increased in response to iodine treatment compared to untreated lettuce leaves.

Furthermore, in a study conducted by Germ et al. [35], buckwheat microgreens (*Fagopyrum esculentum*) were grown for 14 days in plastic plates and irrigated with a nutrient solution containing iodine (1000 mg L^−1^). The microgreens’ yield was reduced by 30% when treated with iodine compared to the control group, which received water irrigation. The reduction in yield was attributed to a phytotoxic effect resulting from excessive iodine absorption by the plants.

In the study by Puccinelli et al. [36], mature lettuce plants irrigated with 10 µM of potassium iodide (KI) in the nutrient solution showed a reduction of approximately 23.9% in dry biomass, 19.9% in fresh biomass, and 20.3% in leaf area. According to the authors, this reduction in biomass was attributed to the accumulation of iodine in the leaves, which caused toxicity and negatively affected gas exchanges and the photosynthetic apparatus.

In a separate experiment conducted by Kiferle et al. [37] using *Arabidopsis thaliana* L., plants grown in the presence of 0.20 and 10 µM of KI did not exhibit phytotoxic symptoms compared to the control group. Morphological parameters such as diameter and length of rosettes and inflorescence, as well as fresh and dry biomass, and seed production, all increased at doses of 0.20 and 10 µM of KI compared to the control. However, plant growth was reduced at a higher dose of 30 µM of KI in the nutrient solution.

Through genetic analysis, Kiferle et al. [37] revealed numerous iodine-regulated genes in the shoots and roots of *Arabidopsis thaliana* plants treated with 10 µM of KI. These genes were found to be up- or down-regulated, and the proteins encoded by these genes were distributed across various cell compartments, including the cell wall, apoplast, vacuoles, cytoplasm, chloroplasts, mitochondria, and nucleus. The study also showed that iodine preferentially bound to certain amino acids, including Tyr, His, Trp, and Cys. Furthermore, Kiferle et al. [37] demonstrated that the iodine-regulated proteins in *Arabidopsis thaliana* leaves were involved in various processes related to photosynthesis. These proteins functioned as structural components of photosystems II and I, participated in the Calvin cycle, the electron transport chain, and the synthesis of ATP and NADP. 

The effective range of Iodine concentration in a nutrient solution, which brings positive effects to plants, was similar to that of other micronutrients, ranging from 10^2^–10^4^ nM [37]. The binding of iodine to proteins in *Arabidopsis thaliana* L. further confirms the role of iodine as an essential plant nutrient [37]. In plants, iodide can also bind to organic molecules such as salicylic acid (iodosalicylates), benzoic acid (iodobenzoates), tyrosine (monoiodotyrosine (MIT), di-iodotyrosine (DIT)), and thyronine (triiodothyronine, T3). However, the metabolic role of these molecules in plants is not yet fully understood. Numerous studies conducted on mature vegetables demonstrated the effectiveness of iodine biofortification in various horticultural species. Examples include lettuce [32], celery (*Apium graveolens* L.) and cabbage (*Brassica oleracea* L.) [38], radish (*Raphanus sativus* L.) [39], spinach (*Spinacia oleracea* L.) [40,41], tomato (*Solanum lycopersicum* L.) [42,43], and carrot (*Daucus carota* L.) [44]. 

### 3.2. Effect of Iodine Biofortification on Microgreens Iodine Concentration

In our study on microgreens, all four species show increased iodine concentration with the biofortification treatment compared to the control group (Figure 1). Among the microgreens, tatsoi had the highest iodine concentration, followed by purple basil. Coriander and green basil had significantly lower iodine values (Figure 1). The study by Blasco et al. [32] also demonstrated efficient iodine transfer and accumulation in mature lettuce leaves at all considered concentrations of iodine, with the highest values observed at a concentration of 10 µM in the nutrient solution. Above this concentration, iodine concentration in plants was reduced [32]. The cultivation system can also influence the biofortification of plants with iodine. In the study by Puccinelli et al. [36], the biofortification of sweet basil and baby-leaf lettuce with 10 µM of KI was influenced by the cultivation system, with a higher iodine concentration observed in plants grown in aeroponics compared to the floating system. In basil plants treated with KI, iodine concentration ranged from 9.76 to 23.58 mg kg^−1^ fw without a reduction in fresh biomass, with the highest values obtained in the aeroponic system. For lettuce, KI intake resulted in an iodine concentration ranging from 1.55 (floating system) to 3.60 mg kg^−1^ fw (aeroponics). Low iodine uptake in plants can be attributed to the volatilization of iodine in the form of methyl iodide (CH_3_I) through stomata, which is facilitated by halide ion methyltransferase (HMT) and halide/thiol methyltransferase (HTMT) enzymes [37]. Another factor contributing to low iodine uptake is the inhibition of absorption by roots, either due to high iodine concentration in the solution surrounding the roots or saturation of transporters [32]. 

### 3.3. Effect of Iodine Biofortification on Pigments and Antioxidants Activities

Carotenoids, such as lutein and β-carotene, are important constituents of thylakoid membranes in plants and play a role in protecting cells against photooxidative damage caused by excessive energy [45]. These pigments contribute to the nutraceutical profile of fruits and vegetables due to their radical scavenging properties [46]. Lutein is particularly beneficial in humans, as it helps prevent macular degeneration [47], while β-carotene serves as a precursor for retinol and vitamin A, which are essential for immune function and vision [48]. In our study on microgreens, there were significant differences in pigment concentration among the different species. Chlorophyll concentration ranges from 1.20 mg 100 g^−1^ fw in coriander to 0.57 mg 100 g^−1^ fw in green basil. Carotenoid concentration also shows statistical differences, with purple basil having the highest lutein and total carotenoid concentration, and coriander having the highest β-carotene concentration. Green basil and tatsoi had the lowest β-carotene and total carotenoid concentration (Figure 2B,D). The iodine treatment does not significantly alter the total carotenoid concentration of tatsoi microgreens. In coriander, the 8 µM treatment increased carotenoid concentration, while in green basil, carotenoid levels were reduced regardless of the dose, compared to the control. In purple basil, the 4 µM dose had a reducing effect on lutein (Figure 2C). In a study on biofortified buckwheat microgreens treated with I (1000 mg L^−1^), there was an increase in chlorophyll a, b, and total carotenoid concentration compared to the control group [35]. Kohlrabi plants biofortified with iodide at a concentration of 8 mM, 21 days after transplantation, showed lower carotenoid and chlorophyll concentration compared to control plants [28]. In the work by Puccinelli et al. [36], both basil and lettuce biofortified with 10 µM of KI exhibited a 20% increase in chlorophyll concentration and a 20.8% reduction in total carotenoid concentration.

Biofortification with iodine had been shown to positively impact selected bioactive compound concentration and antioxidant activity of vegetables. Studies indicate that iodine biofortification can increase glucose, fructose, total sugars [49], and vitamin C concentration in carrot [50]. Additionally, biofortified lettuce with iodine demonstrated an increase in the total concentration of phenols, flavonoids, anthocyanins, ascorbic acid, and total antioxidant capacity [32]. The antioxidant activity of vegetables plays a crucial role in reducing inflammation and combating chronic diseases in humans, such as Alzheimer’s and Parkinson’s, which are associated with oxidative stress caused by reactive oxygen species (ROS). Reactive oxygen species can damage lipids, proteins, DNA, and other macromolecules. Kiferle et al. [37] demonstrated that iodine-regulated genes in *Arabidopsis* plants grown with 10 µM of KI were involved in defense mechanisms against biotic and abiotic stress, regulated antioxidant activity, and participated in the synthesis of enzymes such as peroxidase, oxidase, glutathione S-transferases, and cytochrome P450. These genes were also associated with the metabolism of salicylic acid, a signaling molecule involved in defense against infections. These findings highlight the potential role of low doses of iodine in activating the defense systems of plants. In our study, coriander exhibits the highest antioxidant activity measured by the ABTS method, while green basil and purple basil demonstrate the highest antioxidant activity measured by the FRAP and DPPH methods. Tatsoi had the lowest antioxidant power among the microgreen species tested. Treatment with iodine increases antioxidant activity, as measured by all three methods, in the examined species compared to the untreated control (Figure 3).

The studies conducted by Incrocci et al. [51], Kiferle et al. [52], and Puccinelli et al. [36] on basil treated with iodine (KI) show similar results regarding the increase in antioxidant activity. These studies found a positive correlation between iodine biofortification and the synthesis of polyphenols and essential oils, which contribute to the enhanced antioxidant activity in basil plants. In addition to basil, Blasco et al. [53] demonstrated that increasing the dose of KI (20, 40, 80 µmol L^−1^) led to an increase in the concentration of antioxidant molecules such as ascorbic acid and glutathione, as well as the activity of antioxidant enzyme catalase. However, the activity of superoxide dismutase (SOD), an enzyme involved in ROS scavenging, was reduced, resulting in the accumulation of the radical O_2_ in the leaves and a phytotoxic effect, leading to reduced biomass at high doses of iodine. 

### 3.4. Effect of Iodine Biofortification on Phenolics

Regarding phenolic compounds, the analyses performed in our study using Q Exactive Orbitrap LC–MS/MS revealed significant interactions among the factors considered, indicating the versatility of different species for microgreen production and the importance of agricultural strategies such as biofortification to obtain food products with enhanced nutraceutical characteristics. Phenolic compounds are crucial antioxidant compounds found in fruits and vegetables, and their consumption is associated with reduced risk of diseases caused by oxidative stress. By combating ROS generation, these bioactive molecules are crucial for the proper maintenance and functioning of the human immune system, which, especially in an unknown environment such as space, can be seriously compromised [54]. In our study, green basil and purple basil exhibited higher total phenolic acid concentration, while tatsoi had the lowest values. The different microgreen species were distinguished by their phenolic acid concentration (Table 3). Iodine biofortification had a significant effect on the phenolic acid concentration of all four microgreen species, with specific compounds showing increases compared to the control. For example, in tatsoi, caffeic acid hexoside, coumaroyl quinic acid, and sinapinic acid hexose increased with iodine treatment, while in coriander, feruloyl quinic acid concentration increased. Both basil species showed increased levels of rosmarinic acid with iodine treatments (S × B interaction, Table 2).

Flavonoids are antiallergenic, antiviral, and have antioxidant activities. Their antioxidant potential is related to their high number of hydroxyl groups [32]. In our work, coriander was found to be particularly rich in flavonoids, and in the presence of Iodine, the flavonoid concentration increases, compared to the control (Table 3). Coriander also had a high rutin concentration. Instead, km 3-sinapoyl-sophoroside-7-glucoside was the highest flavonoid in tatsoi. Apigenin-7-rutinoside and km 3-p-coumaroylsophoroside-7-glucoside were the most abundant flavonoids in green and purple basil, respectively (Table 3). The presence of iodine statistically influences the concentration of different flavonoids, with a genotype-dependent effect (Table 3). In the work by Blasco et al. [32], the concentration of 120 and 160 µM of I^−^ increased the flavonoid concentration by four to five times in treated plants than in control plants. The authors also found an increase in the concentration of anthocyanins and vitamin C in plants treated with different doses of I^−^, compared to control plants, reaching the highest concentrations for both molecules at 80 µM of I^−^. In our work, green basil and purple basil had the highest polyphenolic profile, followed by coriander and tatsoi (Table 3). A similar trend was found in the four microgreens species in Pannico et al. [26], where green and purple basil had the highest total polyphenol values (13.698 µg g^−1^ dw and 10.830 µg g^−1^ dw, respectively), followed by coriander (10.237 µg g^−1^ dw) and tatsoi with the lowest values (594 µg g^−1^ dw) (Table 3). Xiao et al. [55] reported a polyphenol range of 1500–7000 μg g^−1^ DW for several microgreen families grown in peat. In our work, iodine treatment increased the total polyphenol concentration in the four species, compared to the control (Table 3). An increase in phenols was observed in lettuce and sweet basil plants exposed to toxic concentrations of iodine (greater than 10 µM or 100 µM, respectively) [32,51,52,56]. The concentration of phenols did not change in iodine-biofortified carrot and tomato, examined by Smoleń et al. [44] and Smoleń et al. [57], respectively, with respect to the control.

## 4. Materials and Methods

### 4.1. Plant Material and Growth Conditions

The four microgreen species, evaluated for their nutraceutical profile and bioactive molecule concentration, belonged to three plant families: *Brassicaceae* (tatsoi, *Brassica rapa* L. subsp. Narinosa), *Apiaceae* (coriander, *Coriandrum sativum* L.), and *Lamiaceae* (green and purple basil, *Ocimum basilicum* L.). Microgreen seedlings were provided by Pagano Costantino and F.lli S.R.L., in Scafati, Salerno, Italy (green basil), Condor Seed Production, Yuma, AZ, USA (purple basil and tatsoi), and Micro Splits, CN Seeds Ltd., Pymoor, Ely, Cambrigeshire, UK (coriander). The four species of microgreens were grown at the Department of Agricultural Sciences of the University of Naples Federico II, in Portici, Italy, in a KBP-6395F growth chamber (Termaks, Bergen, Norway), equipped with a light-emitting system diode) (K5 Series XL750, Kind LED, Santa Rosa, CA, USA). The light spectrum in the 400–700 nm range, and with an intensity of 300 ± 10 µmol m^−2^ s^−1^, at the level of the microgreens canopy. The microgreen seeds were sown in plastic trays (14 W, 19 L, 6 D, cm) on the Capillary Matting substrate (SEAFLO^®^, AP Lifting Gear Company Ltd., Dudley, London, UK) at the density of 7 seeds cm^−2^ for tatsoi, 4 seeds cm^−2^ for coriander, and 6 seeds cm^−2^ for green and purple basil. The trays were rotated by hand inside the growth chamber every day to ensure a homogeneous distribution of light, while the use of a spectral radiometer (MSC15, Gigahertz-Optik, Turkenfeld, Germany) allowed adjusting the luminous spectrum and the photosynthetic photon flux density (PPFD) on the canopy plant. The day/night temperature and relative air humidity in the growth chamber were established at 30/18 °C and 60–70/80%, respectively, with a photoperiod of 12/12 h. A modified quarter-strength Hoagland nutrient solution was used daily to irrigate the plants using a beaker and being careful to evenly wet the substrate. The composition of the solution (in distilled water) was as follows: 2.0 mM nitrate, 0.25 mM sulphur, 0.20 mM phosphorus, 0.62 mM potassium, 0.75 mM calcium, 0.17 mM magnesium, 0.25 mM ammonium, 20 µM iron, 9 µM manganese, 0.3 µM copper, 1.6 µM zinc, 20 µM boron, and 0.3 µM molybdenum. The electrical conductivity of the solution was 0.3 dS m^−1^, and the pH was 6.0. The collection of tatsoi microgreens took place 14 days after sowing (DAS), while that of coriander and basil microgreens was 21 DAS. The experiment was carried out according to a randomized design with two factors, two concentrations (4 µM and 8 µM) of potassium iodide KI (Sigma-Aldrich, St. Louis, MO, USA) in the nutrient solution in addition to an untreated control, and four microgreens’ genotypes (tatsoi, coriander, green basil, purple basil). Each experimental unit was replicated three times, accounting for 36 experimental units in total.

### 4.2. Plant Material Collection, Yield, and Hypocotyl Length 

The harvest of the plants took place when the second true leaf appeared, cutting the stems near the substrate with sterilized scissors. A total of 10 microgreens per replicate were assessed for their hypocotyl length. A part of the fresh material was immediately weighed to determine the yield [Kg of fresh weight (fw) m^−2^] of the 4 species. Fresh samples of each treatment were frozen in liquid nitrogen, stored at –80 °C, and then lyophilized (freeze drier Christ, Alpha 1–4, Osterode, Germany).

### 4.3. Iodine Concentration Determination 

All reagents were purchased from Sigma-Aldrich, with a degree of purity suitable for trace metal analysis and were used as purchased: KI (>99.5%), H_2_O_2_ (50%), NH_4_OH (28%), Na_2_S_2_O_3_ (>98%). All solutions were prepared in MilliQ ultrapure water obtained with a Millipore Plus system (Milano, Italia, resistivity 18 M Ohm cm^−1^). The certified standard SRM 1549a whole milk powder was used to evaluate the performance of the procedure.

Samples of tatsoi, coriander, green basil, and purple basil were frozen, freeze-dried, and pulverized in a porcelain mortar and stored on ice prior to ion chromatography (IC) analysis. For each plant matrix, three samples each were analyzed for control and treatment at 4 and 8 µM (36 samples in total). Each analysis was performed twice to obtain a total of 72 samples. Samples must be prepared for analysis within 24 h to prevent loss of iodide by oxidation.

A Metrohm 940 Professional IC Vario system (Metrohm AG, Herisau, Switzerland) was used to determine the iodide in the plant material. The iodide is separated from the other matrix anions on the Metrosep A Supp 17–100/4.0 mm column with L91 packing, which was identified as the most suitable for the separation of iodide. The chromatographic conditions were as follows: detection: conductivity detection after suppression; temperature: 45 °C; flow rate: 1.0 mL min^−1^; injection volume: 20 μL

Eluent: 10 mM Na_2_CO_3_; run time: 10 min.

Each sample was fully digested in a vessel microwave system MLS-1200 Microwave Lab System (Milestone, Shelton, CT, USA). Approximately 500 mg of each sample were weighed inside the vessels and 8 mL of 50% H_2_O_2_, mixed with 500 µL of 28% NH_4_OH, were added to the vessel containing the sample. Then the microwave system was pressurized. The following microwave heating program was applied: 10 min of ramp and hold for 20 min at temperatures 250 °C. After cooling, the sample digests were stabilized with NH_4_OH and Na_2_S_2_O_3_, then diluted with water up to 25 mL and filtered. The filtrate was analyzed directly by ion chromatography.

A stock solution of 1.0 g L^−1^ of potassium iodide (KI) was prepared by dissolving 1.31 g in 1000 mL of MilliQ ultrapure water. Intermediate working solutions, 100 mg L^−1^ KI, were prepared by pipetting 10 mL of stock solution and diluting to a total volume of 100 mL. Calibration standards between 0.5 mg L^−1^ and 20 mg L^−1^ were prepared by appropriate dilution of the intermediate stock solution with MilliQ ultrapure water. The linearity of iodide was investigated over this concentration range. The correlation coefficient was found to be 0.999. The specificity was checked with the diluent, resolution solution, standard solution, and sample solution to ensure that there was no interference or co-elution with the iodide peak. NIST SRM 1549a was also used to determine the percent recovery. The NIST target value was 3.34 mg kg^−1^ for the iodine analyte, and the analyses averaged 96.6% recovery in 20 separate measurements SRM 1549a whole milk powder.

### 4.4. Chlorophyll and Carotenoids Concentration

The total chlorophyll concentration was determined by extracting 1 g of each fresh microgreen sample from 25 mL of 90% acetone. After a 15 min reaction in the dark, the samples were centrifuged (2000 rpm), and the absorbance of the supernatant was read at wavelengths of 663 and 647 nm (Hach DR 2000 spectrophotometer (Hach Co., Loveland, CO, USA), corresponding to the wavelengths of chlorophyll a and b, respectively. The total chlorophyll concentration was determined using the formulas and extinction coefficients proposed by Lichtenthaler and Buschmann [58]. Data were expressed in mg 100 g^−1^ fw.

For the determination of carotenoids, 100 mg of each lyophilized sample was dissolved in a mixture containing 6 mL of ethanol and 0.1% butylated hydroxytoluene (BHT), as described in Kyriacou et al. [15]. A 20 µL aliquot of the mixture was injected into a Shimadzu HPLC (Model LC 10, Shimadzu, Osaka, Japan) equipped with a reverse phase column (250 × 4.6 mm, 5 µm Gemini C18, Phenomenex, Torrance, CA, USA), obtaining the separation of carotenoids through a gradient run of 25 min. The concentration of the different carotenoids was obtained by constructing a calibration curve obtained from standard solutions of lutein and β-carotene (5–100 µg mL^−1^). All results were expressed as mg 100 g^−1^ fw. 

### 4.5. Antioxidant Activity 

The antioxidant activity was evaluated after extraction of the microgreens with methanol and using the Hach DR 2000 UV–vis spectrophotometry spectrophotometer (Hach Co., Loveland, CO, USA) according to protocols described by Formisano et al. [59]. The 2,2-diphenyl-1-picrylhydrazyl (DPPH) method involved incubation of 200 µL of extract from each microgreen sample, at room temperature for 10 min with 1 mL of DPPH solution (4 mg of DPPH in 10 mL of methanol) and reading the extracts at 517 nm. 

Ferric reducing antioxidant activity (FRAP method) involved incubation for 4 min at room temperature of 150 µL of microgreen extract with 2.850 mL of FRAP working solution (1.25 mL of 10 mM 2,4,6- tripyridyl-striazine (TPTZ) in HCl (40 mM), 1.25 mL of FeCl3 (20 mmol) in water and 12.5 mL of 0.3 M sodium acetate buffer 0.3 M (pH 3.6). The absorbance of the samples was read at a length of 593 nm, thanks to the reduction in ferric tripyridyltriazine [Fe (III)-TPTZ] to colored ferrous tripyridyltriazine [Fe (II)—TPTZ]. 

The 2,2-azinobis-(3-ethylbenzothiazoline-6-sulfonate) (ABTS) method involved incubation for three minutes of 100 µL of each sample extract with 1 mL of a solution containing ABTS^+^ radicals (5 mL of 7 mM ABTS aqueous solution, 88 µL of 2.45 mM potassium persulfate and ethanol). The absorbance of the samples was carried out at 734 nm. The results of the three methods were expressed in mmol Trolox (6-hydroxy-2,5,7,8-tetramethylchro man-2-carboxylic acid) equivalent kg^−1^ of dry weight (dw) of the sample, based on an external calibration curve (0–250 µM) built using Trolox as standard.

### 4.6. Determination of Polyphenols

One hundred mg of each lyophilized sample was used to determine the polyphenols concentration, as described in Kyriacou et al. [15]. The samples were analyzed with a UHPLC system (Thermo Fisher Scientific, Waltham, MA, USA), equipped with a quaternary pump (Ultimate 3000 Dionex, Sunnyvale, CA, USA) and a thermostated column (100 × 2.1 mm, Kinetex 1.7 µm biphenyl, Phenomenex, Torrance, CA, USA). Mass spectrometry analysis was performed using a Q Exactive Orbitrap LC–MS/MS (Thermo Fisher Scientific, Waltham, MA, USA). The determination of the polyphenol concentrations was possible thanks to a reference standard mixture (Thermo Fisher Scientific, Waltham, MA, USA). Data analysis was performed with Xcalibur software, version 3.0.63 (Thermo Fisher Scientific, Waltham, MA, USA). All results were expressed as µg 100 g^−1^ fw. 

### 4.7. Statistics

Data were subjected to a two-way analysis of variance (ANOVA). Interactions were subjected to genotype-specific one-way analysis of variance. Treatment means have been compared according to Tukey’s HSD test (*p* = 0.05), through the SPSS 20 software package (IBM, Armonk, NY, USA).

## 5. Conclusions

The psychophysical health of astronauts is of utmost importance for the success of long-duration Space missions and planetary colonization. Microgreens, with their excellent nutritional profile and adaptability to Space cultivation systems, have recently been introduced as potential food garnishing for future astronauts. The integration of agronomic biofortification strategies further enhances the cultivation of microgreens in Space. Our results indicate that, regardless of the species, biofortification with iodine in the nutrient solution increases the levels of this micronutrient in plant tissues. A portion (10 g) of tatsoi microgreens bio-enriched with 8 µM of iodine would cover the recommended daily dose (150 µ day^−1^) of this trace element of astronauts. However, it is observed that the biofortification strategy leads to a reduction in fresh yield and an increase in phenolic and flavonoid acids in a genotype-dependent manner. On the other hand, the ABTS antioxidant activity increases in all genotypes, irrespective of the iodine dose used. When implementing biofortification strategies, it is crucial to consider the variability induced by different genotypes. This is important to ensure that the purpose of biofortification, which is not only to enrich the crop with micronutrients but also to improve the nutraceutical profile without significant yield reductions, is effectively achieved. Taking into account genotype-induced variability is essential to support the prolonged stay of astronauts on Space missions or stations, where their psychophysical health is paramount.

## Figures and Tables

**Table 1:** Fresh biomass (kg m^−2^), dry biomass (kg m^−2^), and hypocotyl length (cm) of four microgreen genotypes grown with three concentrations of iodine in the nutrient solution.

Treatment	Fresh Biomass	Dry Biomass	Hypocotyl Length
	kg m^−2^		cm
Species (S)			
Tatsoi	1.52 ± 0.03 b	0.11 ± 0.00 b	3.18 ± 0.03 c
Coriander	1.78 ± 0.03 a	0.20 ± 0.00 a	4.30 ± 0.06 a
Green basil	1.10 ± 0.03 c	0.10 ± 0.00 c	3.79 ± 0.03 b
Purple basil	1.04 ± 0.02 d	0.09 ± 0.00 d	4.24 ± 0.08 a
	***	***	***
Biofortification (B)			
Control	1.44 ± 0.09 a	0.13 ± 0.01 a	3.91 ± 0.16
4 µM	1.31 ± 0.10 b	0.12 ± 0.01 b	3.81 ± 0.13
8 µM	1.34 ± 0.09 b	0.12 ± 0.01 b	3.90 ± 0.14
	***	***	n.s.
S × B			
Tatsoi × control	1.60 ± 0.01 c	0.12 ± 0.00	3.17 ± 0.03 d
Tatsoi × 4 µM	1.39 ± 0.04 d	0.11 ± 0.00	3.13 ± 0.10 d
Tatsoi × 8 µM	1.58 ± 0.01 c	0.12 ± 0.00	3.23 ± 0.03 d
Coriander × control	1.85 ± 0.02 a	0.20 ± 0.00	4.20 ± 0.12 ab
Coriander × 4 µM	1.81 ± 0.06 ab	0.19 ± 0.01	4.21 ± 0.03 ab
Coriander × 8 µM	1.69 ± 0.04 bc	0.19 ± 0.00	4.49 ± 0.09 a
Green basil × control	1.20 ± 0.03 e	0.11 ± 0.00	3.76 ± 0.05 c
Green basil × 4 µM	1.03 ± 0.03 f	0.10 ± 0.00	3.79 ± 0.01 c
Green basil × 8 µM	1.09 ± 0.00 ef	0.10 ± 0.00	3.81 ± 0.10 c
Purple basil × control	1.10 ± 0.01 ef	0.09 ± 0.00	4.52 ± 0.06 a
Purple basil × 4 µM	0.99 ± 0.01 f	0.09 ± 0.00	4.11 ± 0.09 bc
Purple basil × 8 µM	1.02 ± 0.01 f	0.09 ± 0.00	4.09 ± 0.04 bc
	***	n.s.	**

n.s., **, *** Non-significant or significant at *p* ≤ 0.01 and 0.001, respectively. Different letters within each column indicate significant mean differences among the genotypes according to Tukey’s HSD test (*p* = 0.05). All data are expressed as mean ± SE, *n* = 3.

**Table 2:** Total phenolic acids and phenolic acids concentration of four microgreens genotypes grown with three concentrations of iodine in the nutrient solution.

Treatment	Caffeic Acid Hexoside	Coumaroyl Quinic Acid	Sinapinic Acid Hexose	Feruloyl Quinic Acid	Caffeoyl Quinic Acid	Caffeic Acid
	µg 100 g^−1^ fw					
Species (S)						
Tatsoi	85.26 ± 5.43 a	21.84 ± 1.58 a	168.24 ± 4.76 a	5.38 ± 0.34 a	10.12 ± 0.60 b	4.02 ± 0.22 c
Coriander	9.72 ± 0.45 b	0.17 ± 0.04 b	17.45 ± 0.67 b	662.51 ± 17.73 c	3600.43 ± 182.27 a	6.15 ± 0.27 c
Green basil	9.03 ± 0.61 b	0.22 ± 0.02 b	16.4 ± 0.54 b	6.33 ± 0.53 c	62.38 ± 10.07 b	36.20 ± 2.81 a
Purple basil	13.54 ± 0.34 b	0.23 ± 0.03 b	9.55 ± 0.23 c	48.95 ± 1.91 b	18.30 ± 0.95 b	25.11 ± 0.97 b
	***	***	***	***	***	***
Biofortification (B)						
Control	25.71 ± 7.66 b	4.42 ± 2.21 b	51.18 ± 19.28 b	167.73 ± 76.75 b	926.29 ± 464.57	16.13 ± 3.36
4 µM	32.40 ± 11.57 a	6.09 ± 3.12 b	57.50 ± 22.43 a	187.52 ± 88.09 a	868.86 ± 449.57	18.31 ± 4.53
8 µM	30.05 ± 10.31 ab	8.39 ± 4.14 a	50.05 ± 18.62 b	187.13 ± 87.64 a	973.27 ± 501.64	19.17 ± 4.65
	*	*	***	*	n.s.	n.s.
S × B						
Tatsoi × control	69.25 ± 4.40 b	16.92 ± 1.70 b	161.79 ± 1.79 b	4.45 ± 0.32 c	11.65 ± 0.70	4.24 ± 0.53 c
Tatsoi × 4 µM	97.85 ± 9.23 a	23.82 ± 2.06 a	186.18 ± 2.30 a	6.25 ± 0.42 c	10.29 ± 0.63	4.28 ± 0.33 c
Tatsoi × 8 µM	88.66 ± 5.99 a	24.78 ± 2.12 a	156.74 ± 3.93 b	5.44 ± 0.56 c	8.43 ± 0.88	3.52 ± 0.14 c
Coriander × control	9.18 ± 0.81 c	0.25 ± 0.01 c	16.14 ± 0.61 cd	607.27 ± 8.16 b	3574.32 ± 266.25	6.03 ± 0.26 c
Coriander × 4 µM	9.47 ± 1.14 c	0.09 ± 0.00 c	18.14 ± 1.74 c	691.84 ± 25.46 a	3401.9 ± 410.60	5.77 ± 0.77 c
Coriander × 8 µM	10.51 ± 0.17 c	nd	18.08 ± 0.86 c	688.41 ± 27.70 a	3825.07 ± 337.51	6.63 ± 0.20 c
Green basil × control	10.95 ± 0.25 c	0.29 ± 0.02 c	17.21 ± 0.99 cd	7.17 ± 1.09 c	98.91 ± 13.69	27.60 ± 2.10 b
Green basil × 4 µM	9.11 ± 0.13 c	0.13 ± 0.00 c	16.52 ± 0.34 cd	6.99 ± 0.68 c	46.91 ± 2.68	39.28 ± 5.64 a
Green basil × 8 µM	7.03 ± 0.70 c	0.25 ± 0.00 c	15.47 ± 1.30 cd	4.81 ± 0.05 c	41.33 ± 3.60	41.73 ± 1.33 a
Purple basil × control	13.45 ± 0.70 c	0.23 ± 0.00 c	9.58 ± 0.33 d	52.01 ± 4.13 c	20.28 ± 0.35	26.65 ± 0.55 b
Purple basil × 4 µM	13.18 ± 0.29 c	0.33 ± 0.01 c	9.17 ± 0.35 d	45.00 ± 2.88 c	16.34 ± 1.15	23.89 ± 2.58 b
Purple basil × 8 µM	14.00 ± 0.79 c	0.14 ± 0.00 c	9.91 ± 0.49 cd	49.85 ± 2.30 c	18.26 ± 2.37	24.79 ± 1.52 b
	**	**	***	**	n.s.	**
**Treatment**	**Ferulic Acid**	**Rosmarinic Acid**	**Caffeoyl–Feruloyl–Tartaric Acid**		**Cichoric Acid**	**Total Phenolic Acids**
			**µg 100 g^−1^ fw**			
Species (S)						
Tatsoi	0.97 ± 0.03 d	nd	nd		nd	295.82 ± 9.80 c
Coriander	65.74 ± 2.02 a	nd	nd		nd	4362.10 ± 180.80 b
Green basil	14.37 ± 1.33 b	14,696.01 ± 606.65	325.85 ± 15.49 a		1249.37 ± 105.63	16,416.16 ± 609.75 a
Purple basil	6.95 ± 0.38 c	14,274.07 ± 776.5	199.22 ± 9.25 b		1135.39 ± 99.57	15,731.32 ± 798.21 a
	***	n.s.	***		n.s.	***
Biofortification (B)						
Control	22.12 ± 7.80	12,613.59 ± 179.26 b	281.66 ± 35.25		1335.59 ± 127.16	8328.99 ± 1880.87
4 µM	22.19 ± 7.42	14,945.96 ± 1028.18 a	240.86 ± 27.82		1097.97 ± 119.92	9335.28 ± 2240.83
8 µM	21.70 ± 8.20	15,895.57 ± 356.20 a	265.10 ± 31.65		1143.58 ± 124.94	9939.79 ± 2312.86
	n.s.	**	n.s.		n.s.	*
S × B						
Tatsoi × control	1.03 ± 0.07	nd	nd		nd	269.33 ± 6.56 d
Tatsoi × 4 µM	0.90 ± 0.05	nd	nd		nd	329.57 ± 9.61 d
Tatsoi × 8 µM	0.98 ± 0.03	nd	nd		nd	288.56 ± 8.34 d
Coriander × control	65.76 ± 4.34	nd	nd		nd	4278.96 ± 261.22 c
Coriander × 4 µM	63.18 ± 3.29	nd	nd		nd	4190.39 ± 385.82 c
Coriander × 8 µM	68.27 ± 3.58	nd	nd		nd	4616.96 ± 352.85 c
Green basil × control	15.04 ± 0.49	12,780.35 ± 293.32	355.25 ± 26.44		1431.68 ± 200.64	14,744.46 ± 491.6 ab
Green basil × 4 µM	18.47 ± 0.57	16,063.09 ± 1111.59	294.38 ± 30.83		1171.19 ± 235.18	17,666.06 ± 1313.64 a
Green basil × 8 µM	9.59 ± 0.72	15,244.58 ± 418.17	327.93 ± 17.38		1145.25 ± 115.00	16,837.97 ± 476.27 ab
Purple basil × control	6.65 ± 0.80	12,446.82 ± 216.39	208.06 ± 9.89		1239.51 ± 177.09	14,023.23 ± 217.80 b
Purple basil × 4 µM	6.22 ± 0.47	13,828.84 ± 1673.96	187.34 ± 7.42		1024.76 ± 106.00	15,155.07 ± 1781.08 ab
Purple basil × 8 µM	7.97 ± 0.23	16,546.56 ± 189.04	202.26 ± 27.56		1141.90 ± 254.60	18,015.65 ± 438.35 a
	n.s.	n.s.	n.s.		n.s.	*

n.s., *, **, *** Non-significant or significant at *p* ≤ 0.05, 0.01, and 0.001, respectively. Different letters in each column indicate significant mean differences among the genotypes according to Tukey’s HSD test (*p* = 0.05). All data are expressed as mean ± SE, *n* = 3. nd: not detected. fw: fresh weight.

**Table 3:** Total polyphenols and flavonoids concentration of four microgreens genotypes grown with three iodine concentrations in the nutrient solution.

Treatment	Luteolin-7-O-glucoside	Qn 3-sophoroside-7-glucoside	Km 3-sophoroside-7-glucoside	Qn 3-caffeoyl-sophoroside-7-glucoside	Km 3-sinapoyl-sophoroside-7-glucoside	Km 3-sinapoyl-sophorotrioside-7-glucoside	Km 3-O-caffeoyl-spophoroside-7-glucoside	Quercetin-3-sophoroside	Isorhamnetin-3-gentiobioside
	µg 100 g^−1^ fw								
Species (S)									
Tatsoi	nd	0.40 ± 0.03	1.6 ± 0.15 a	6.14 ± 0.49 b	88.29 ± 2.35 a	0.47 ± 0.06	36.7 ± 2.06 a	1.34 ± 0.18 b	4.94 ± 0.18
Coriander	nd	nd	1.00 ± 0.18 b	29.76 ± 1.74 a	0.71 ± 0.11 b	nd	nd	2.05 ± 0.18 a	nd
Green basil	1.37 ± 0.15 a	nd	nd	nd	nd	nd	nd	nd	nd
Purple basil	0.86 ± 0.11 b	nd	nd	nd	1.97 ± 0.12 b	nd	0.57 ± 0.05 b	1.22 ± 0.13 b	nd
	***	-	**	***	***	-	***	**	-
Biofortification (B)									
Control	1.58 ± 0.17 a	0.32 ± 0.03 b	1.23 ± 0.33	16.63 ± 5.73	32.47 ± 15.65	0.33 ± 0.01 b	15.59 ± 6.70 b	1.61 ± 0.23	5.26 ± 0.23
4 µM	0.95 ± 0.09 b	0.41 ± 0.02 ab	1.17 ± 0.22	18.60 ± 5.70	29.20 ± 13.98	0.41 ± 0.01 b	19.45 ± 8.60 ab	1.43 ± 0.20	4.49 ± 0.28
8 µM	0.81 ± 0.11 b	0.48 ± 0.03 a	1.49 ± 0.14	18.62 ± 5.06	29.30 ± 13.94	0.67 ± 0.07 a	20.85 ± 9.17 a	1.58 ± 0.19	5.07 ± 0.33
	***	*	n.s.	n.s.	n.s.	**	*	n.s.	n.s.
S×B									
Tatsoi × control	nd	nd	1.92 ± 0.17 a	4.75 ± 0.33 b	95.04 ± 1.27 a	nd	30.57 ± 0.54 b	0.97 ± 0.11	nd
Tatsoi × 4 µM	nd	nd	1.50 ± 0.35 abc	6.00 ± 0.44 b	85.01 ± 3.18 b	nd	38.37 ± 3.49 ab	1.38 ± 0.42	nd
Tatsoi × 8 µM	nd	nd	1.37 ± 0.23 abc	7.67 ± 0.63 b	84.81 ± 4.49 b	nd	41.15 ± 2.88 a	1.68 ± 0.25	nd
Coriander × control	nd	nd	0.55 ± 0.23 c	28.51 ± 4.81 a	0.44 ± 0.20 c	nd	nd	2.38 ± 0.17	nd
Coriander × 4 µM	nd	nd	0.85 ± 0.08 bc	31.21 ± 1.89 a	0.72 ± 0.13 c	nd	nd	1.90 ± 0.25	nd
Coriander × 8 µM	nd	nd	1.61 ± 0.17 ab	29.57 ± 2.79 a	0.97 ± 0.13 c	nd	nd	1.87 ± 0.48	nd
Green basil × control	1.94 ± 0.02	nd	nd	nd	nd	nd	nd	nd	nd
Green basil × 4 µM	1.11 ± 0.09	nd	nd	nd	nd	nd	nd	nd	nd
Green basil × 8 µM	1.04 ± 0.01	nd	nd	nd	nd	nd	nd	nd	nd
Purple basil × control	1.22 ± 0.14	nd	nd	nd	1.92 ± 0.12 c	nd	0.62 ± 0.07 c	1.48 ± 0.32	nd
Purple basil × 4 µM	0.79 ± 0.05	nd	nd	nd	1.88 ± 0.32 c	nd	0.53 ± 0.14 c	1.00 ± 0.13	nd
Purple basil × 8 µM	0.58 ± 0.07	nd	nd	nd	2.11 ± 0.24 c	nd	0.55 ± 0.07 c	1.19 ± 0.16	nd
	n.s.	-	*	n.s.	*	-	*	n.s.	-
**Treatment**	**Km 3-feruloylsophoroside-7-glucoside**	**Hyperoside**	**Rutin**	**Quercetin-3-glucoside**		**Km 3-p-coumaroylsophoroside-7-glucoside**		**Luteolin-malonil-hexose**	**Quercetin Rhamnoside**
				**µg 100 g^−1^ fw**					
Species (S)									
Tatsoi	13.4 ± 0.55	nd	12.86 ± 0.56 b	nd		12.95 ± 1.10 b		0.13 ± 0.01 a	0.71 ± 0.06 c
Coriander	nd	26.92 ± 1.86 a	6430.29 ± 292.91 a	20.58 ± 1.46 a		nd		0.08 ± 0.01 bc	0.46 ± 0.13 c
Green basil	nd	1.48 ± 0.13 c	1.77 ± 0.63 b	1.37 ± 0.12 c		nd		nd	12.37 ± 0.65 a
Purple basil	nd	18.07 ± 0.59 b	2.66 ± 0.13 b	16.91 ± 0.56 b		31.87 ± 1.30 a		nd	8.89 ± 0.30 b
	-	***	***	***		***		***	***
Biofortification (B)									
Control	12.19 ± 0.96	14.89 ± 3.53	1347.60 ± 701.30 c	12.79 ± 2.94 ab		21.89 ± 5.86		0.13 ± 0.02 a	5.57 ± 1.47
4 µM	14.55 ± 0.78	14.13 ± 3.53	2224.83 ± 1110.47 a	11.45 ± 2.71 b		22.83 ± 3.78		0.10 ± 0.02 b	5.38 ± 1.59
8 µM	13.45 ± 0.88	17.46 ± 4.45	1819.02 ± 947.73 b	14.62 ± 3.44 a		22.51 ± 3.46		0.09 ± 0.01 b	5.87 ± 1.70
	n.s.	n.s.	***	*		n.s.		**	n.s.
S × B									
Tatsoi × control	nd	nd	11.29 ± 0.78 c	nd		9.10 ± 1.19 b		0.17 ± 0.02 a	0.53 ± 0.02
Tatsoi × 4 µM	nd	nd	14.52 ± 0.55 c	nd		14.61 ± 0.43 b		0.14 ± 0.00 a	0.66 ± 0.01
Tatsoi × 8 µM	nd	nd	12.78 ± 0.49 c	nd		15.15 ± 1.35 b		0.08 ± 0.01 bc	0.95 ± 0.03
Coriander × control	nd	25.74 ± 1.77	5373.4 ± 123.58 c	21.2 ± 1.91 ab		nd		0.08 ± 0.00 bc	0.97 ± 0.02
Coriander × 4 µM	nd	23.18 ± 4.03	6656.93 ± 255.3 b	16.50 ± 2.49 b		nd		0.06 ± 0.01 c	0.21 ± 0.01
Coriander × 8 µM	nd	31.83 ± 1.48	7260.54 ± 142.22 a	24.03 ± 1.08 a		nd		0.10 ± 0.01 b	0.20 ± 0.02
Green basil × control	nd	1.87 ± 0.13	3.17 ± 0.20 c	1.72 ± 0.09 c		nd		nd	11.13 ± 0.81
Green basil × 4 µM	nd	1.13 ± 0.08	nd	1.03 ± 0.04 c		nd		nd	12.24 ± 1.55
Green basil × 8 µM	nd	1.44 ± 0.21	0.37 ± 0.02 c	1.36 ± 0.19 c		nd		nd	13.74 ± 0.56
Purple basil × control	nd	17.05 ± 0.43	2.55 ± 0.17 c	15.46 ± 0.38 b		34.67 ± 2.58 a		nd	9.66 ± 0.32
Purple basil × 4 µM	nd	18.08 ± 0.53	3.05 ± 0.22 c	16.81 ± 0.60 b		31.05 ± 1.91 a		nd	8.41 ± 0.64
Purple basil × 8 µM	nd	19.09 ± 1.62	2.39 ± 0.03 c	18.45 ± 0.99 ab		29.87 ± 1.94 a		nd	8.60 ± 0.38
	-	n.s.	***	*		*		***	n.s.
**Treatment**	**Apigenin-malonil-glucoside**	**Apigenin-7-rutinoside**	**Kaempferol-rutinoside**	**Kaempferol-glucoside**	**Apigenin-7-rhamnoside-4-rutinoside**	**Apigenin-7-glucoside**	**Luteolin-7-rutinoside**	**Total Flavonoids**	**Total Polyphenols**
	**µg 100 g^−1^ fw**								
Species (S)									
Tatsoi	0.38 ± 0.03 c	0.87 ± 0.12 c	0.49 ± 0.08 b	0.35 ± 0.04 c	2.59 ± 0.24	nd	nd	184.62 ± 4.66 b	480.44 ± 12.73 c
Coriander	nd	1.35 ± 0.43 c	43.55 ± 1.55 a	0.19 ± 0.02 c	nd	nd	51.00 ± 2.18 a	6607.93 ± 296.30 a	10,970.03 ± 375.70 b
Green basil	6.25 ± 0.39 a	18.57 ± 0.83 b	2.33 ± 0.17 b	8.60 ± 0.29 a	nd	3.63 ± 0.18 a	0.23 ± 0.03 b	57.38 ± 0.88 b	16,473.54 ± 610.04 a
Purple basil	1.94 ± 0.18 b	21.44 ± 0.84 a	2.95 ± 0.18 b	5.81 ± 0.24 b	nd	1.29 ± 0.09 b	1.84 ± 0.23 b	118.30 ± 2.20 b	15,849.61 ± 799.34 a
	***	***	***	***	-	***	***	***	***
Biofortification (B)									
Control	3.51 ± 1.10 a	9.56 ± 2.41 b	11.68 ± 5.14	3.70 ± 1.06	2.14 ± 0.19 b	2.34 ± 0.46	15.37 ± 7.40 b	1473.35 ± 708.96 b	9802.34 ± 1733.69 b
4 µM	2.32 ± 0.75 c	10.59 ± 3.03 ab	13.03 ± 5.84	3.71 ± 1.11	2.13 ± 0.15 b	2.37 ± 0.54	17.72 ± 8.35 ab	1796.75 ± 877.88 a	11,132.02 ± 2040.28 a
8 µM	2.74 ± 0.80 b	11.52 ± 3.22 a	12.28 ± 5.44	3.80 ± 1.12	3.51 ± 0.15 a	2.67 ± 0.64	19.98 ± 9.34 a	1956.06 ± 957.39 a	11,895.85 ± 2108.70 a
	***	***	n.s.	n.s.	***	n.s.	**	***	***
S × B									
Tatsoi × control	0.29 ± 0.04 e	0.46 ± 0.03 d	0.25 ± 0.02	0.23 ± 0.00 d	2.14 ± 0.19	nd	nd	175.81 ± 3.30 d	445.13 ± 5.21
Tatsoi × 4 µM	0.37 ± 0.02 e	0.85 ± 0.02 d	0.45 ± 0.07	0.34 ± 0.05 d	2.13 ± 0.15	nd	nd	186.19 ± 8.44 d	515.77 ± 17.74
Tatsoi × 8 µM	0.47 ± 0.01 e	1.30 ± 0.09 d	0.77 ± 0.07	0.49 ± 0.01 d	3.51 ± 0.15	nd	nd	191.85 ± 10.57 d	480.42 ± 18.87
Coriander × control	nd	3.05 ± 0.12 d	40.97 ± 2.60	0.24 ± 0.01 d	nd	nd	44.79 ± 2.94 b	5542.3 ± 128.63 c	9821.26 ± 382.44
Coriander × 4 µM	nd	0.36 ± 0.06 d	46.27 ± 3.23	0.15 ± 0.01 d	nd	nd	50.93 ± 2.78 ab	6829.27 ± 257.49 b	11,019.66 ± 180.17
Coriander × 8 µM	nd	0.64 ± 0.01 d	43.41 ± 2.12	0.16 ± 0.02 d	nd	nd	57.27 ± 1.25 a	7452.21 ± 148.27 a	12,069.17 ± 499.91
Green basil × control	7.71 ± 0.21 a	15.55 ± 0.38 c	2.77 ± 0.16	7.88 ± 0.41 ab	nd	3.36 ± 0.08	0.33 ± 0.02 c	57.44 ± 1.07 d	14,801.90 ± 492.56
Green basil × 4 µM	5.23 ± 0.34 b	19.77 ± 1.06 b	2.12 ± 0.26	8.84 ± 0.52 a	nd	3.50 ± 0.37	0.23 ± 0.01 c	55.21 ± 1.74 d	17,721.28 ± 1315.35
Green basil × 8 µM	5.81 ± 0.10 b	20.38 ± 0.33 b	2.08 ± 0.35	9.08 ± 0.39 a	nd	4.03 ± 0.38	0.13 ± 0.01 c	59.48 ± 0.76 d	16,897.45 ± 477.02
Purple basil × control	2.53 ± 0.15 c	19.18 ± 0.74 b	2.73 ± 0.39	6.46 ± 0.33 bc	nd	1.31 ± 0.10	1.01 ± 0.11 c	117.85 ± 2.85 d	14,141.08 ± 216.17
Purple basil × 4 µM	1.35 ± 0.13 d	21.37 ± 1.05 ab	3.26 ± 0.27	5.50 ± 0.40 c	nd	1.24 ± 0.22	1.99 ± 0.08 c	116.32 ± 2.89 d	15,271.39 ± 1783.96
Purple basil × 8 µM	1.95 ± 0.13 cd	23.77 ± 1.26 a	2.85 ± 0.28	5.47 ± 0.28 c	nd	1.31 ± 0.19	2.53 ± 0.05 c	120.72 ± 6.07 d	18,136.36 ± 443.46
	***	***	n.s.	*	-	n.s.	**	***	n.s.

n.s., *, **, *** Non-significant or significant at *p* ≤ 0.05, 0.01, and 0.001, respectively. Different letters in each column indicate significant mean differences among the genotypes according to Tukey’s HSD test (*p* = 0.05). All data are expressed as mean ± SE, *n* = 3. Nd: not detected. fw: fresh weight.

## Data Availability

The data are contained within the article.

## Supplementary Materials

The following supporting information can be downloaded at: https://www.mdpi.com/article/10.3390/plants12142628/s1, Table S1: Pigments and antioxidant activity of four microgreen genotypes grown with three concentrations of iodine in the nutrient solution.
