# Supplementary material for: Iodine-Biofortified Microgreens as High Nutraceutical Value Component of Space Mission Crew Diets and Candidate for Extraterrestrial Cultivation
Source: Plants (Basel). 2023 Jul 12;12(14):2628. doi: 10.3390/plants12142628 (PMC10384207; doi:10.3390/plants12142628)
Supplement: Supplementary file 1 [file plants-12-02628-s001.zip › plants-2485125-supplementary.pdf]

plants

MDPI

Supplementary Table S1. Pigments and antioxidant activity of four microgreen genotypes grown with three concentrations of iodine in the nutrient solution.

Treatment	Total chlorophyll	β-carotene	Lutein	Total carotenoids	ABTS	DPPH	FRAP
	mg 100 g^{-1} fw				mmol Trolox equivalents kg^{-1} dw.		
Species (S)							
Tatsoi	1.20 ± 0.04a	139.83 ± 4.33d	54.94 ± 2.06c	194.77 ± 6.26c	93.57 ± 1.18d	94.79 ± 2.18d	158.63 ± 3.19d
Coriander	1.04 ± 0.04b	347.44 ± 12.29a	91.72 ± 4.11b	439.16 ± 16.11a	156.21 ± 1.99a	204.80 ± 9.52c	276.91 ± 10.05b
Green Basil	0.57 ± 0.04c	179.72 ± 9.68c	84.95 ± 4.43b	264.67 ± 14.01b	143.49 ± 2.87b	213.59 ± 3.21b	281.88 ± 3.07a
Purple Basil	0.67 ± 0.03c	278.20 ± 3.84b	143.45 ± 3.46a	421.66 ± 6.57a	136.14 ± 2.92c	220.86 ± 3.79a	269.41 ± 8.33c
	***	***	***	***	***	***	***
Biofortification (B)							
Control	0.89 ± 0.08	240.94 ± 18.84a	98.21 ± 10.69a	339.15 ± 25.84a	124.69 ± 6.58b	167.78 ± 14.59c	226.66 ± 14.28c
4 µM	0.88 ± 0.09	222.84 ± 25.64b	88.03 ± 9.62b	310.88 ± 32.99b	135.68 ± 7.09a	188.83 ± 15.00b	251.41 ± 14.78b
8 µM	0.83 ± 0.08	245.11 ± 30.47a	95.06 ± 9.68a	340.17 ± 37.56a	136.69 ± 7.83a	193.92 ± 17.81a	262.06 ± 18.22a
	n.s.	***	***	***	***	***	***
S×B							
Tatsoi × Control	1.17 ± 0.06	151.11 ± 4.45fg	58.73 ± 0.45ef	209.84 ± 4.75de	89.63 ± 0.68j	88.86 ± 1.37g	147.63 ± 1.10j
Tatsoi × 4 µM	1.29 ± 0.09	124.75 ± 2.86g	47.10 ± 1.98f	171.85 ± 4.83e	97.60 ± 0.57h	102.92 ± 1.10f	169.32 ± 1.61h
Tatsoi × 8 µM	1.14 ± 0.04	143.62 ± 3.55fg	58.99 ± 0.69ef	202.61 ± 4.23de	93.47 ± 0.06i	92.59 ± 0.85fg	158.95 ± 0.56i
Coriander × Control	1.10 ± 0.07	313.38 ± 4.08bc	79.23 ± 1.63d	392.61 ± 3.09b	149.02 ± 0.17d	168.78 ± 4.59e	239.41 ± 3.17g
Coriander × 4 µM	1.02 ± 0.05	337.47 ± 4.92b	92.72 ± 4.46cd	430.19 ± 9.36b	156.94 ± 0.37b	213.79 ± 1.87c	283.80 ± 0.78cd
Coriander × 8 µM	1.00 ± 0.10	391.47 ± 13.16a	103.22 ± 5.97c	494.69 ± 18.74a	162.68 ± 0.39a	231.82 ± 2.85ab	307.52 ± 2.59a
Green Basil × Control	0.67 ± 0.06	218.12 ± 2.43e	101.70 ± 3.44c	319.82 ± 5.30c	133.36 ± 0.26f	202.15 ± 2.05d	272.72 ± 1.10e
Green Basil × 4 µM	0.55 ± 0.05	160.61 ± 3.40f	77.62 ± 3.05d	238.22 ± 6.21d	153.12 ± 0.41c	222.64 ± 2.13bc	292.80 ± 2.90bc
Green Basil × 8 µM	0.48 ± 0.04	160.44 ± 1.27f	75.54 ± 1.73de	235.98 ± 2.71d	143.99 ± 0.95e	216.00 ± 2.38c	280.13 ± 0.81de
Purple Basil × Control	0.63 ± 0.06	281.17 ± 6.07d	153.18 ± 4.74a	434.34 ± 10.81b	126.74 ± 0.68g	211.33 ± 0.30cd	246.88 ± 1.17g
Purple Basil × 4 µM	0.66 ± 0.07	268.55 ± 7.84d	134.69 ± 3.49b	403.23 ± 10.55b	135.05 ± 0.85f	216.00 ± 0.59c	259.72 ± 3.23f
Purple Basil × 8 µM	0.71 ± 0.02	284.90 ± 2.30cd	142.49 ± 4.78ab	427.39 ± 4.76b	146.63 ± 1.19de	235.26 ± 3.25a	301.65 ± 0.76ab
	n.s.	***	***	***	***	***	***

n.s., *** Non significant or significant at $p < 0.001$, respectively. Different letters in each column indicate significant mean differences among the genotypes according to Tukey's HSD test ($p = 0.05$). All data are expressed as mean ± SE, $n = 3$.
